# A Molecular Epidemiological and Genetic Diversity Study of Tuberculosis in Ibadan, Nnewi and Abuja, Nigeria

**DOI:** 10.1371/journal.pone.0038409

**Published:** 2012-06-18

**Authors:** Lovett Lawson, Jian Zhang, Michel K. Gomgnimbou, Saddiq T. Abdurrahman, Stéphanie Le Moullec, Fatima Mohamed, Gertrude N. Uzoewulu, Olumide M. Sogaolu, Khye Seng Goh, Nnamdi Emenyonu, Guislaine Refrégier, Luis E. Cuevas, Christophe Sola

**Affiliations:** 1 Zankli Medical Centre, Abuja, Nigeria; 2 Institut de Génétique et Microbiologie UMR8621, CNRS-Université Paris-Sud, Orsay, France; 3 National Tuberculosis and Leprosy Control Programme, Abuja, Nigeria; 4 Nnamdi Azikiwe Teaching Hospital, Nnewi, Nigeria; 5 University College Hospital, Ibadan, Nigeria; 6 Tuberculosis Laboratory Consultant, Les Abymes, Guadeloupe, France; 7 Liverpool School of Tropical Medicine, Liverpool, United Kingdom; St. Petersburg Pasteur Institute, Russian Federation

## Abstract

**Background:**

Nigeria has the tenth highest burden of tuberculosis (TB) among the 22 TB high-burden countries in the world. This study describes the biodiversity and epidemiology of drug-susceptible and drug-resistant TB in Ibadan, Nnewi and Abuja, using 409 DNAs extracted from culture positive TB isolates.

**Methodology/Principal Findings:**

DNAs extracted from clinical isolates of *Mycobacterium tuberculosis* complex were studied by spoligotyping and 24 VNTR typing. The Cameroon clade (CAM) was predominant followed by the *M. africanum* (West African 1) and T (mainly T2) clades. By using a smooth definition of clusters, 32 likely epi-linked clusters related to the Cameroon genotype family and 15 likely epi-linked clusters related to other “modern” genotypes were detected. Eight clusters concerned *M. africanum* West African 1. The recent transmission rate of TB was 38%. This large study shows that the recent transmission of TB in Nigeria is high, without major regional differences, with MDR-TB clusters. Improvement in the TB control programme is imperative to address the TB control problem in Nigeria.

## Introduction

Multi-drug-resistant *Mycobacterium tuberculosis* (MDR-TB) has emerged as a major global public health problem [1]. WHO estimates that in 2008, between 390,000 and 510,000 persons developed MDR-TB worldwide with 69,000 cases occurring in Africa and 11,000 in Nigeria [2]. Nigeria has the tenth highest burden of TB among the 22 TB high-burden countries and an estimated TB incidence rate of 320/100000 population (WHO 2011). MDR-TB is an emerging problem in Nigeria with as much as 8% of all cultured specimens being MDR-TB in Ibadan, Nnewi and Abuja [2].

Despite the ever growing importance of TB in Nigeria, available molecular epidemiological studies do not represent an extensive picture of TB epi-links in this country due to non-standard genotyping protocols and restricted sampling areas [3]–[6]. This is due to molecular diagnostic methods being until now poorly adapted to high TB prevalence due to high costs or suboptimal protocols to ensure epi-links detection. Hence, the African TB molecular epidemiology is poorly described with the exception of South Africa [7], [8].

Recent innovations in molecular diagnostics (e.g. Hain MTBDR*plus*®, MTBDR*sl* ®, GenXpert®) and genotyping procedures such as the analysis of 24 Mycobacterial Interspersed Repetitive Units-Variable Number of Tandem Repeats (MIRU-VNTR) and high-throughput spoligotyping have made the analysis of TB transmission more efficient and the MDR-TB diagnostics easier [9], [10]. Multiplexed high-throughput technologies are also emerging as powerful tools both for molecular diagnostics and public health with whole genome sequencing (WGS) holding promise for this field [11], [12]. 24 MIRU-VNTR combined with spoligotyping is a new standard to replace the IS*6110*-Restriction Fragment Length Polymorphism (RFLP) fingerprinting method. Analyzing hundreds and even thousands of clinical isolates’ DNA with limited resources has now become feasible [13], [14].

Combining spoligotyping and MIRU-VNTR allows to analyze the genetic diversity and molecular epidemiology of drug susceptible and MDR-TB strains with the aim to identify the population structure of circulating clinical isolates, to estimate the recent TB transmission rate, and to eventually detect the transmission of MDR-TB cases [2].

In this manuscript, we present the characterization of the drug-susceptible and MDR-TB *M. tuberculosis* isolates previously reported by Lawson’s et *al.* (2011) in Nigeria using molecular markers and we estimate the recent TB transmission rate in Nigeria [2]. We also identify the main clades of circulating genotypes.

## Materials and Methods

### Biological Samples, Clinical Isolates, Drug Susceptibility Testing, DNA Extraction

Sputum specimens were obtained through a cross-sectional study aiming at describing TB drug resistance in three cities of Nigeria. These are cities situated in three different geopolitical zones of Nigeria, as reported in Lawson *et al.*, 2011 [2]. Five hundred twenty patients were recruited (520), including 433 newly diagnosed patients with pulmonary TB (PTB) and 87 patients with PTB who had failed to respond to first-line TB treatment attending TB diagnostic centres in three different geopolitical zones of Nigeria. Patients over 18 years old with positive smear microscopy attending Directly Observed Treatment Short course (DOTS) treatment centres were enrolled prospectively from August 2009 to July 2010 at 1) Wuse, Maitama, Asokoro and Nyanya General Hospitals in Abuja, 2) the University College Hospital and five DOTS centres in Ibadan and 3) Nnamdi Azikiwe Teaching Hospital and three DOTS centres in Nnewi. Clinical features of these specimens were described in Lawson *et al.* [2]. The protocol for the study received Ethical approval from the Federal Capital Territory Health and Research Ethics Committee and Zankli Ethical Research Review Board. Written consent was obtained from all participants including permission to store samples to conduct further tests for characterising the strains affecting them.

Drug Susceptibility Tests were Performed in Liquid (Bactec®MGIT960 System, Becton Dickinson, NJ) Media for 426 Culture-positive *Mycobacterium Tuberculosis Complex* (MTBC) of the 520 Participants (82%) with Smear-positive Sputum Samples.

DNA was extracted from isolates derived from sputum specimens cultured on BACTEC® MGIT960 by a thermolyzate method and sent by express carrier on ice to the Institut de Génétique et Microbiologie UMR8621 CNRS-University Paris-Sud.

### Genotyping Methods, Data Analysis

**Figure 1:**
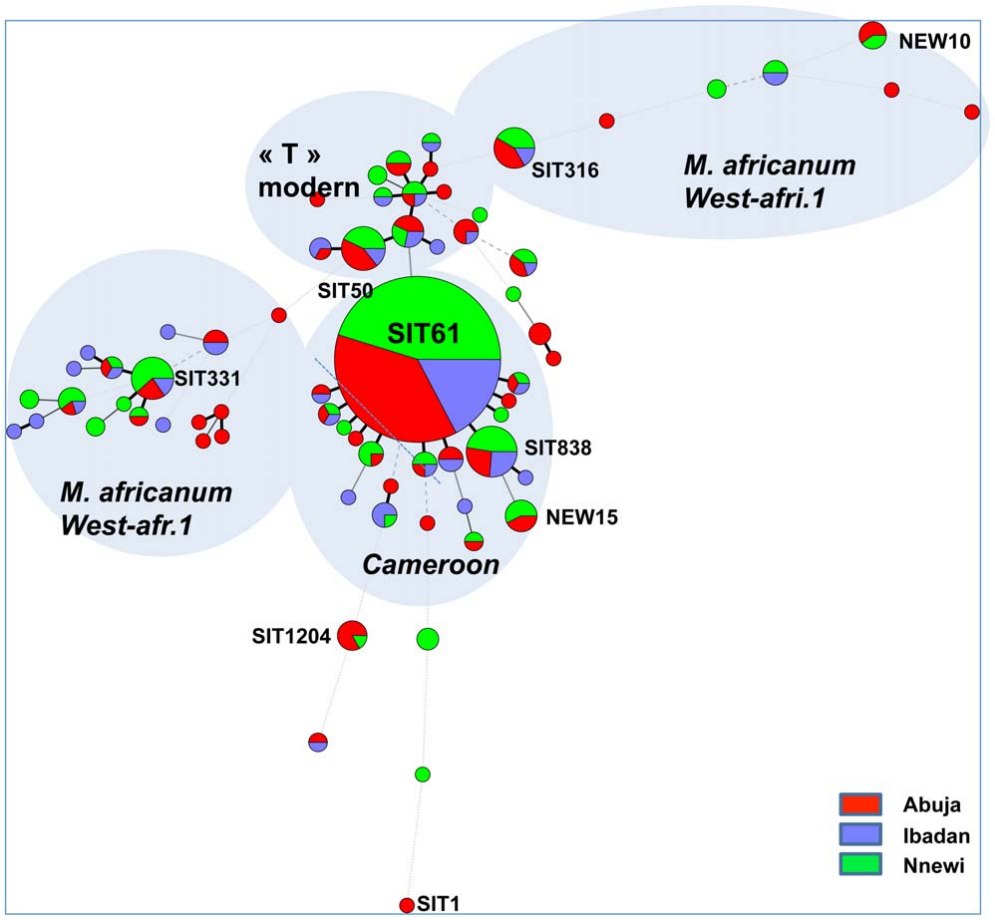
Minimum Spanning Tree (MST) of all available spoligotypes (n = 408; Nnewi n = 172 green colour, Abuja n = 154 red colour, Ibadan, n = 82 blue colour), constructed and drawn using Bionumerics (v.6.6, Applied Maths, Sint-Martens-Latem, Belgium) and the “advanced cluster analysis” method. Some prevalent clades are designated and identified using Spoligotype-International-Types (SIT). Main Clades (Cameroon, *M. africanum* West African 1, modern “T” are also shown.

**Figure 2:**
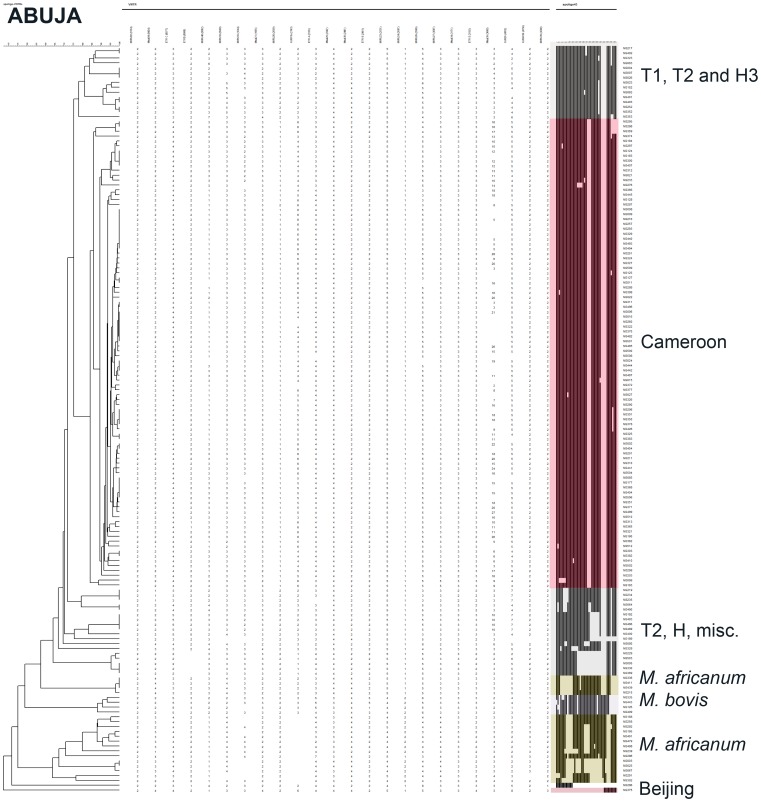
Unweighted Pair Group Method using Mathematical Averages (UPGMA) dendrogram (first column) built with Bionumerics® on a composite data set (24 VNTR-largest column)-Spoligotyping (coloured column) on the clinical isolates from Abuja patients. (identification number : last column) Main Clades are also annotated right to identification number.

**Figure 3:**
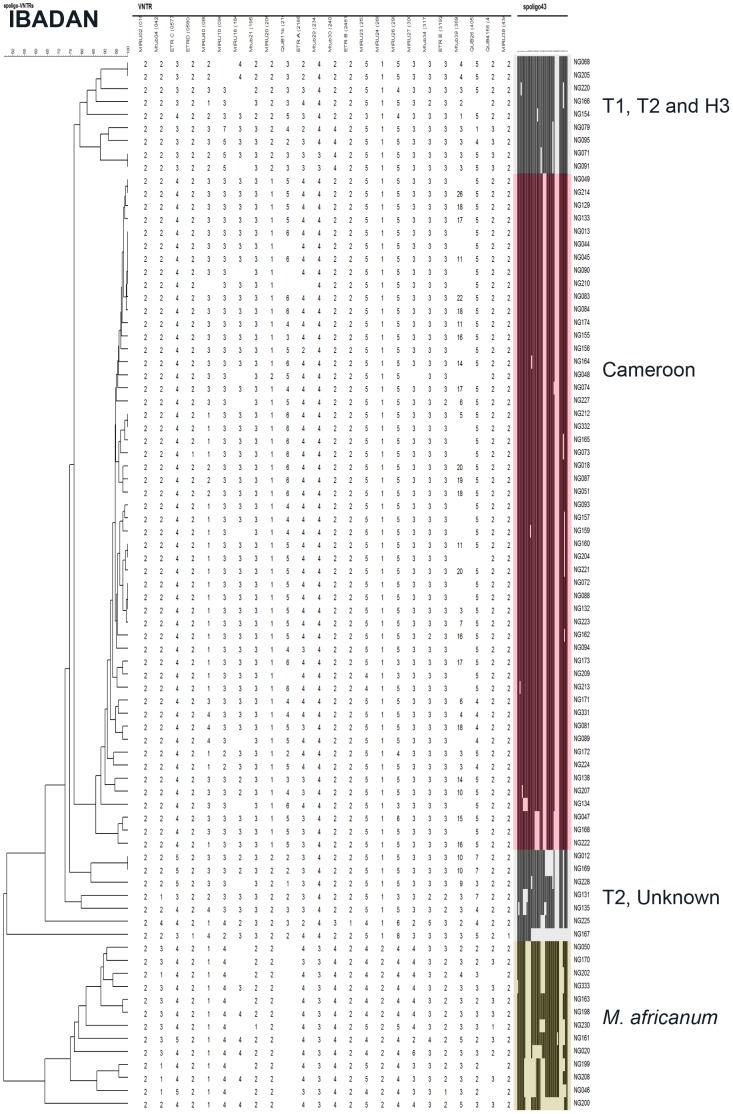
Unweighted Pair Group Method using Mathematical Averages (UPGMA) dendrogram (first column) built with Bionumerics® on a composite data set (24 VNTR-largest column)-Spoligotyping (coloured column) on the clinical isolates from Ibadan patients. (identification number : last column) Main Clades are also annotated right to identification number.

**Figure 4:**
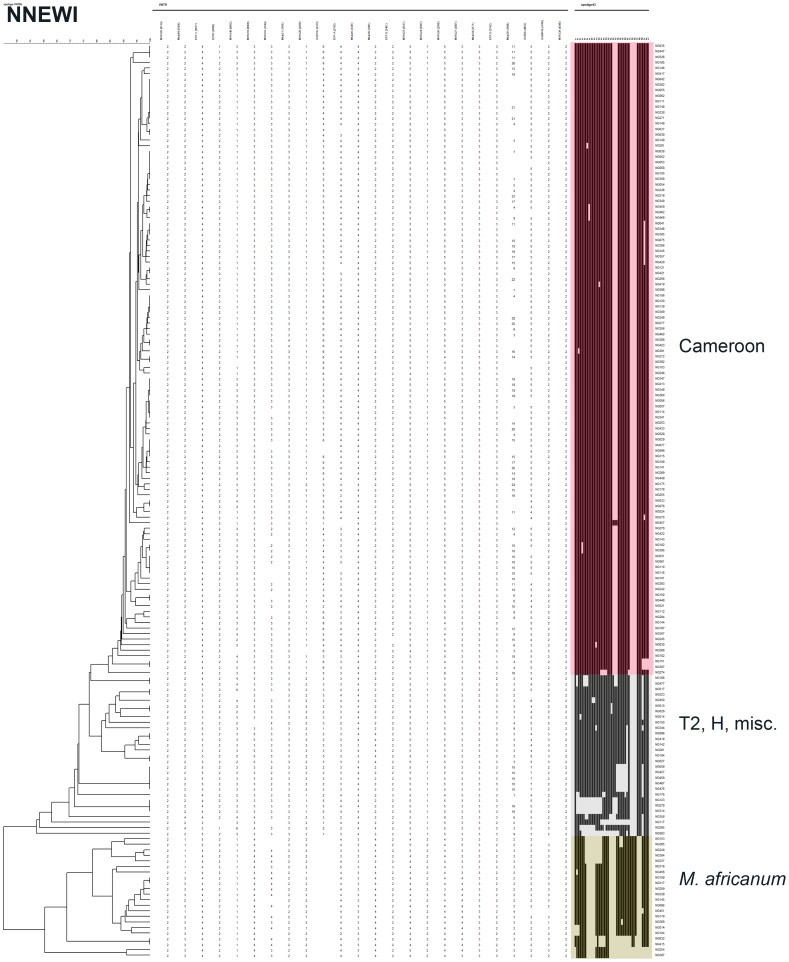
Unweighted Pair Group Method using Mathematical Averages (UPGMA) dendrogram (first column) built with Bionumerics® on a composite data set (24 VNTR-largest column)-Spoligotyping (coloured column) on the clinical isolates from Nnewi patients. (identification number : last column) Main Clades are also annotated right to identification number.

High-throughput spoligotyping was performed on Luminex 200® (Luminex Corp., TX) as previously described [15]. Twenty-four VNTR loci analysis was done on agarose gels after simplex PCR, as previously reported [16], or using an updated in house duplex procedure (Refrégier *et al.* unpublished data). The standardized 24 VNTR loci method was used [10]. DNA was available for 423 isolates (353 from new cases and 70 from previously treated patients); complete genotyping defined as spoligotyping plus at least 20 MIRU-VNTR markers was obtained for 404 samples (97%). From these, 154 were obtained from Abuja (38%), 81 from Ibadan (20%) and 169 from Nnewi (42%). All data were entered into Excel® files and transferred to Bionumerics® (v.6.6 Applied Maths, Sint Martin Latems, Belgium). Cluster analysis was performed in Bionumerics® according to instructions manual (Figure 1, 2, 3, 4). Clade/Family assignation was done based on SpolDB4 for available Spoligo-International-Types (up to SIT1939) and based on SITVITWEB for SIT n° 2088 and 2550 [17], [18]. MIRU-VNTR international type (MIT) designation was done using SITVITWEB [18]. When no SIT n° were available in SpolDB4 or SITVITWEB, the designation “new-x” (lower cases) was given for orphan pattern and the designation “NEW-X” for new intra-study clusters. The recent transmission Index (RTI) was computed using the (n−1) method in which the number of isolates in clusters minus the number of clusters divided by the total number of isolates represents the recent tuberculosis transmission rate; in our case: (RTn-1 = 152–51/410) [19]. Statistical analyses (Odds ratio, Chi-square and Student’s T test and ANOVA) were performed in R version 2.13.0 (www.r-project.org/).

## Results

### Population Description

MTBC strains were isolated from 423 patients (163 men, 253 women, and 7 with unregistered gender). Twenty-three patients were <20 years old, 289 between 20 and 40 years, 87 between 40 and 60 years and 17>60 years old.

### *M. tuberculosis* Complex Genetic Diversity in Nigeria Studied by Spoligotyping

Spoligotyping results (43 spacers format) were obtained for 412 of 423 (97%) isolates (Fig 1, 2, 3, 4 and Table S1). The global population structure is made up of 4 main clusters: one from the Cameroon clade (CAM), two from the *Mycobacterium africanum* West African 1, and one from other “modern” clinical isolates (Figure 1). Seventy-one different spoligotypes were identified (Figures 2, 3, 4 and Table S1). Thirty-five of these patterns were unique and 36 occurred in clusters totaling 377 isolates, containing a range of 2 to 208 isolates. The CAM family (formerly designated as Latin American and Mediterranean-10-CAM or LAM-10-CAM) predominated, with 269 isolates (65%), of which 261 clustered and 8 were orphans). *M. africanum* West African 1 represented 53 isolates (13%) of the isolates with 42 clustered and 11 orphans. Other genotype families were: 1) “modern” strains including T2 (n = 29; 7%), Haarlem (n = 31; 7%), and Latin-American-Mediterranean (LAM) (n = 2; 0.5%), 2) Beijing (n = 1; 0.25%), 3) *Mycobacterium bovis* (n = 4; 1%) and 4) other unclassified isolates (n = 12; 3%). Most CAM family isolates (n = 208, 77%) had the SIT61; 10 other CAM-related spoligotype profiles were identified (Table 1): SIT838 found in 19 isolates (7%), further 4 SIT types containing from 1 to 4 isolates and 6 undesignated types in the SpolDB4 worldwide database [17] shared by 2 to 6 isolates and 7 orphan patterns. The four spoligotype profiles characteristic of *M. bovis* showed the absence of spacer 30 which is a specific characteristic of the “African 1” *M. bovis* genotype family previously described as predominant in Western Central Africa (Nigeria, Cameroun, Mali, Chad) [20]. By comparison with the *M. bovis* database (www.mbovis.org, see also Table 1), three isolates matched with known profiles SB0944 (SIT1037), SB1026 (new-h), SB1099 (new-d), and one isolate (new-b) had not been previously described.

**Table 1:** Main spoligotyping clusters, orphan isolates and new variants within the Cameroon (CAM) (A) *M. bovis* (B), and *M.* *africanum* (C) genotype families found in this study.

	SIT		Spoligotype binary	Spoligotype Octal	n° of Strains
**A**	61		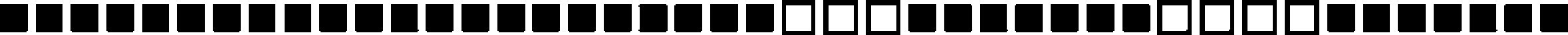	777777743760771	208
	838		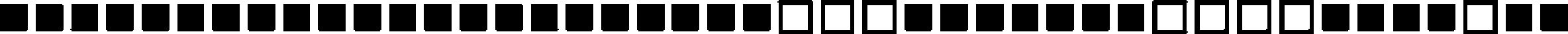	777777743760751	19
	403		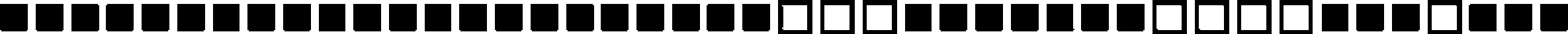	777777743760731	4
	852		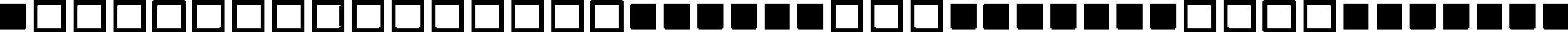	400003743760771	3
	2550		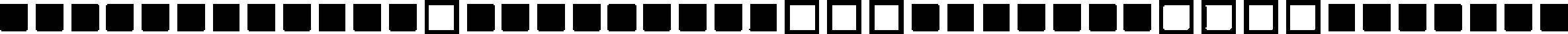	777737743760771	3
	NEW15*		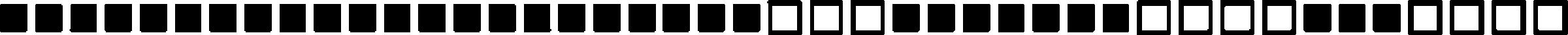	777777743760700	7
	NEW14*		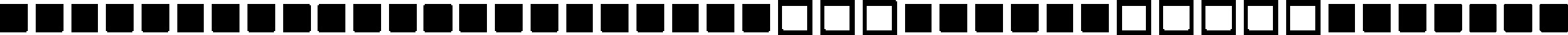	777777743740771	2
	NEW13*		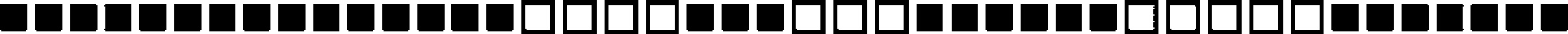	777770343740771	4
	NEW12*		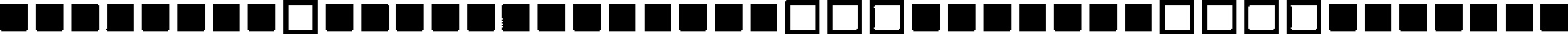	776777743760771	4
	NEW6*		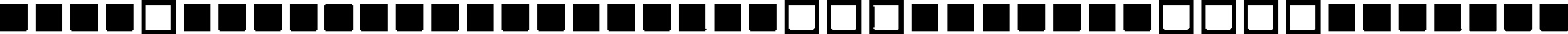	757777743760771	4
	NEW4*		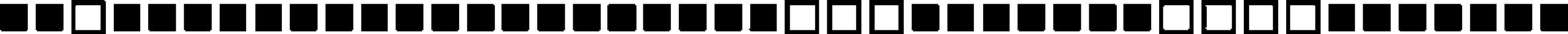	677777743760771	3
**B**	1037		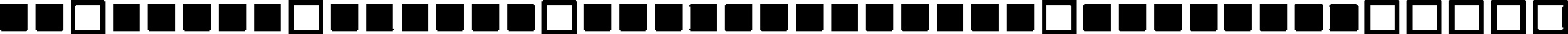	676773777677600	1
	new-h*		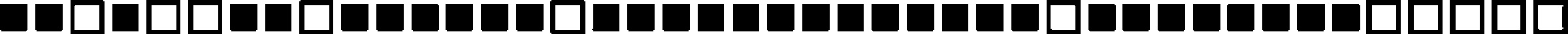	646773777677600	1
	new-d*		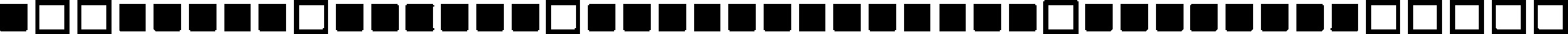	476773777677600	1
	new-b*		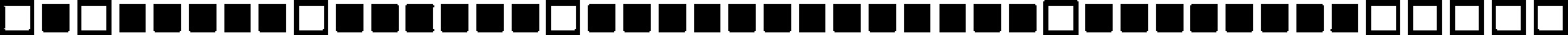	276773777677600	1
**C**	320		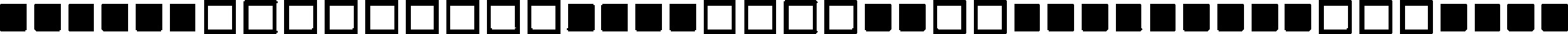	770007414777071	2
	330		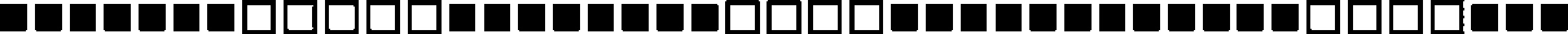	774077607777031	3
	331		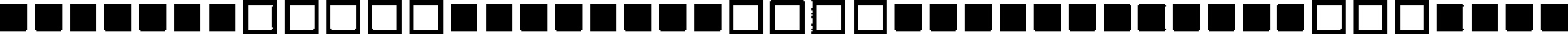	774077607777071	13
	NEW1*		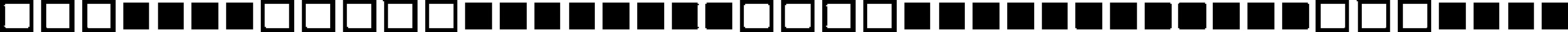	074077607777071	2
	NEW2*		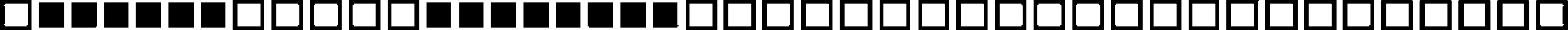	374077600000000	2
	NEW8*		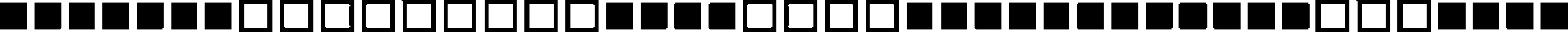	774003607777071	5
	NEW10*		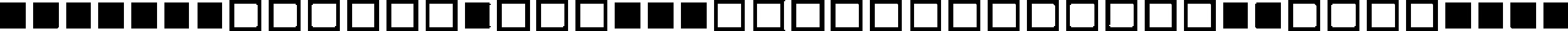	774021600006071	5
	NEW11*	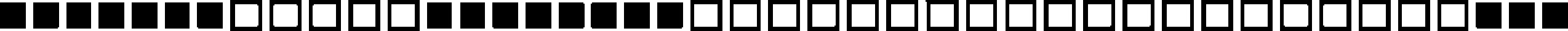		774077600000031	4
	NEW16*	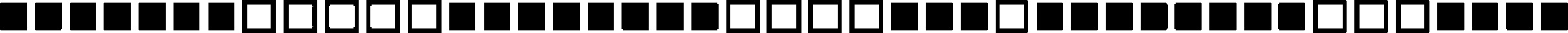		774077607377071	2

In column B, a star (*) appears next to the « NEW » label when no Spoligo-International-Type (SIT) number was available within the international database SpolDB4. SIT1037 is also designated as SB0944 in the M. bovis.org database (SB nomenclature), new-h is identical to SB1026, new-d is identical to SB1099, whereas new-b has no SB number.

53 *M. africanum* isolates were found. 52 lacked spacers 8–12 and 37–39 and thus belonged undoubtedly to *M. africanum* West African 1 (MAF1) [21]. The largest cluster within MAF1 was ST331, with 13 isolates. One isolate from Abuja (“new-s”) exhibited a partial signature of MAF1 (absence of spacers 37–39 but presence of spacers 8 and 10–12 [21]. Spoligotyping on an additional 25 spacers set (68 spacers spoligotyping) unfortunately did not much improve the clusterization of all clinical isolates (Table S1).

### 24 VNTR Results; Recent Transmission Index Assessment

Exploitable VNTR results (defined as less than 4 missing values) were obtained for 404 of 423 isolates (Table S1). Some isolates did not provide results for many loci. Among the most problematic loci, Mtub39 (VNTR3690) characterisation frequently failed (n = 78). The failure to amplify Mtub39 was linked to isolates belonging to the CAM family suggesting an amplification problem specific to this family (Odds Ratio = 4.8; IC_95_ = [2.5; 10.1]). Recent results in Indian isolates have pointed to the large variability of copy number of VNTR3690 [22]. A similar observation was made for the 57 isolates in which there was a failure to amplify QuB11b (VNTR2163b), among which all *M. africanum*. None of the 53 *M. africanum* clinical isolates provided results on QuB11b whereas only 20 among the 370 other strains did not (χ^2^268). As this feature seems to rely on genetic properties of all *M. africanum* isolates, QuB11b marker was hypothesized to be identical (“X” or not determined “ND” in Table S1) for all isolates from this group. Finally, 45 isolates failed to amplify for MIRU16, 23 of which belong to the *M. africanum* and *M. bovis* clade (OR = 6.4; IC_95_ = [3.3; 12.6]).

Sub-populations were tentatively identified among CAM isolates using spoligotyping and 24 VNTR. The identified clusters exhibited 20, 16, 21, 26, 15,16 and 4 clinical isolates respectively (Table 2). A large proportion of CAM isolates could not be amplified for Mtub39 locus. The remaining isolates had a surprisingly diverse copy numbers. The values ranged from 2 to 28 copies and are noted as “N” (Table 2) (cf. also Table S1). Spoligotyping splits the CAM sub-group G-V isolates into two sub-clusters of 15 and 16 isolates. Among “Unknown” isolates according to SpolDB4, six sharing the spoligotype SIT1204 shared 24 VNTR patterns suggesting a phylogenetic link with CAM family. The SIT1204 genotype was already described in the Cross River State in the South geopolitical zone of Nigeria [4]. These strains differed only on the Mtub39 locus and harboured an intermediate copy number (n = 2.5 copies) at the exact tandem repeat D (ETR-D) locus [23]. They may represent an epi-cluster.

**Table 2:** Main sub-clusters according to Multi Locus VNTR and spoligotyping within the «Cameroon» (CAM) clade.

Sub	SIT	Nb	MIRU2	Mtub04	ETRC	ETRD	MIRU40	MIRU10	MIRU16	Mtub21	MIRU20	Qub11b	ETRA	Mtub29	Mtub30	ETRB	MIRU23	MIRU24	MIRU26	MIRU27	Mtub34	ETRE	Mtub39	Qub26	QuB4156	MIRU39	MIRU Int Type
**G-I**	61	20	2	2	4	2	3	3	3	3	1	5	4	4	2	2	5	1	5	3	3	3	N	5	2	2	MIT12
**G-II**	61	16	2	2	4	2	3	3	3	3	1	6	4	4	2	2	5	1	5	3	3	3	N	5	2	2	MIT12
**G-III**	61	21	2	2	4	2	1	3	3	3	1	4	4	4	2	2	5	1	5	3	3	3	N	5	2	2	MIT266
**G-IV**	61	26	2	2	4	2	1	3	3	3	1	6	4	4	2	2	5	1	5	3	3	3	N	5	2	2	MIT266
**G-V-1**	61	15	2	2	4	2	1	3	3	3	1	5	4	4	2	2	5	1	5	3	3	3	N	5	2	2	MIT266
**G-V-2**	838	16	2	2	4	2	1	3	3	3	1	5	4	4	2	2	5	1	5	3	3	3	N	5	2	2	MIT266
**G-VI**	61	4	2	2	4	2	3	3	2	3	1	≤4	3	4	2	2	5	1	5	3	3	3	N	≤5	2	2	MIT264

Sub =  Subgroup name, SIT =  spoligo-International type, Nb =  Number of strains in that group. MIT = MIRU-International Type.

(MIT266 = 36%; MIT12 = 27%, MIT264 = 7%); Mtub 39, N =  high copy number and variable, MIRU16, 40 and QuB11b : poorly variable, all other markers : no variation.

Alias designation of MIRU-VNTR loci (with genomic positions) : VNTR0154 = MIRU2, VNTR0424 = Mtub4, VNTR0577 = ETRC, VNTR0580 = ETRD or MIRU4, VNTR0802 = MIRU40, VNTR0960 = MIRU10, VNTR1644 = MIRU16, VNTR1955 = Mtub21, VNTR2059 = MIRU20, VNTR2163 = QUB11B, VNTR2165 = ETRA, VNTR2347 = Mtub29, VNTR2401 = Mtub30, VNTR2461 = ETRB, VNTR2531 = MIRU23, VNTR2687 = MIRU24, VNTR2996 = MIRU26, VNTR3007 = MIRU27, VNTR3171 = Mtub34, VNTR3192 = ETRE or MIRU31, VNTR3690 = Mtub39, VNTR4052 = QUB26, VNTR4156 = QUB4156, VNTR4348 = MIRU39.

If defining clusters by using 100% identity between isolates according to both spoligotyping and VNTR typing, and considering missing values as unique so that any pattern with a missing value cannot belong to a cluster, 134 isolates were grouped in 47 clusters containing 2 to 11 isolates. If these figures are considered a true representation of the epidemiological situation and using the (n−1) method, the recent TB transmission rate would be around 22% in Nigeria [19]. However when single-variants are included, the number of clusters doubles reaching 219 isolates grouped in 65 clusters and a Recent Transmission Index (RTI) of 38% (Table 3). The analysis using VNTR genotyping data alone did not give significantly lower discriminatory power than the composite one, i.e. adding spoligotyping information (Table 3).

**Table 3:** Clustering Results (global or by location) and Recent Transmission Index (RTI) computed using the (n−1) method and various cluster definitions.

Method	Total isolates	Number of Clusters	Number of Clustered Isolates*	Number of Orphan isolates*	RTI
composite* (100% identity)	404	47	134	270	21.5%
composite* (SLV permitted)	404	65	219	185	38.1%
VNTR (100% identity)	404	51	152	257	25%
Spoligotyping (100% identity)	404	36	379	30	NA**
Abuja-Spoligotyping	154	15	125	29	NA**
Abuja-VNTR	154	24	77	77	34%
Abuja-composite(SLV-permitted)	154	21	70	84	32%
Nnewi-Spoligotyping	169	19	153	16	NA**
Nnewi-VNTR	169	29	90	79	36%
Nnewi-composite(SLV-permitted)	169	28	83	86	33%
Ibadan-Spoligotyping	81	15	58	23	NA**
Ibadan-VNTR	81	9	25	56	19%
Ibadan-composite(SLV-permitted)	81	8	21	60	16%

SLV permitted  =  authorizing VNTR single locus variants (SLV) or one « missing data » locus.

composite =  spoligo+VNTR dataset; patterns with missing data are treated as unique patterns, ** Not applicable.

Amplification for QuB11b did not work for *M. africanum* isolates and clustering analysis was thus conducted without this marker. A high polymorphism of MLVA (multi-locus VNTR analysis) was observed within *M. africanum* clinical isolates DNA with identical spoligotypes, suggesting that spoligotyping-based clustering represented common ancestors with no clear epidemiological links in most cases. Indeed, among the 49 *M. africanum* isolates for which MLVA results were available, 4 clusters only of 2 isolates were identified using a strict cluster definition (100% identity). VNTR clusters were also found in the T and H families (Table S1) with six out of eight SIT53 (T1) isolates found in one single cluster. Amongst the 41 SIT52 or derived types, also designated as Ghana family, and using 100% identity, only 17 isolates were found in 4 clusters (4 SIT2088, 4 SIT846, 5 designated as “NEW3” and 4 designated as “NEW5”). 11 of 13 SIT316 isolates (T2-variant) were found in one cluster, whereas two other isolates differed on only one single VNTR locus, Miru26 [24]. These two isolates are likely to represent a second epidemiologically-linked cluster.

### An Evolutionary Scenario of the “Cameroon” (CAM) Clade

The CAM clade, was first described in Cameroon and shows a typical SIT61 signature [25], [26]. The MLVA analysis of the CAM isolates in Nigeria, provides evolutionary and epidemiologic information and together with the 43 spacers spoligotyping, describes a global population analysis of at least 7 main clusters. The combination of values obtained on MIRU16 and MIRU40 (greyed out numbers in following text) allows the observation of three main MIRU12 international types (MIT) as described in the SITVITWEB database (see http://www.pasteur-guadeloupe.fr:8081/SITVIT_ONLINE): 223315153323, reported as MIRU-international-type 12 (MIT12), 223315153321, reported as MIT266 and 223215153323, reported as MIT264. All three major VNTR 12 types were independently reported in Nigeria in another study [4]. QuB11B provides further epidemiological information within the sub-clades (Table 2). Assuming a molecular evolution by loss of copies on MIRU40, the ancestral character of this marker would be 3 and the ancestral MIT signature would be MIT12, which would have independently evolved in MIT264 and MIT266. The larger diversity observed in Mtub39 for MIT12 and MIT266 (from 5 to 28 copies, see also Table S1) than for MIT264 (from 10 to 12 copies) reinforces this hypothesis.

### Distribution of Multi-Drug Resistance Isolates

Twenty-nine (29 *i.e.* 7%) of 407 isolates with phenotypic Drug Susceptibility Testing (DST) in BACTEC-MGIT® (Becton Dickinson, NJ, USA) were MDR-TB isolates as previously described [2]. Among these, 23 belonged to the CAM family, of which 17 were SIT61 (data not shown). The proportion of MDR-TB within the CAM family is statistically not different from the percentage of the CAM family in the whole population (Student’s test T = 0.1; df = 260; p-value = 0.9). Three MDR-TB isolates belonged to T, one to LAM, one to *M. africanum* and one to U (Unknown). Nine MDR-TB isolates belonged to the subgroup G-III of the CAM family which contains 21 isolates (*i.e.* 43% MDR in this subgroup). Nine additional isolates were resistant to at least one of the drugs tested (altogether 85% of resistance). The CAM and T clades exhibited a high resistance level with respectively 53% and 54% being resistant to at least one drug. Among the 53 *M. africanum* isolates studied, one only was MDR and 17 (32%) were resistant to one of the drugs tested. The proportion of MDR was higher among the H family (28%, 9 out of 32).

### Spatial and Phylogenetical Analysis of Diversity and Transmission

To detect if specific MTBC clusters were circulating in specific geographical areas, cluster analyses were performed independently for each collecting center (Figures 2, 3 and 4 for Abuja, Ibadan, and Nnewi, respectively). The number of clustered isolates of the 3 centers was reduced to 72 as compared to 134 in the complete study (54%) when considering 100% identity, and 144 as compared to 219 (66%) when allowing for inclusion of SLVs. The prevalence of the main clades was similar in the three cities (p = 0.59). These results confirm that Nigeria can be considered as homogeneous in the three settings investigated regarding the origin of isolates. A linear model was searched for to identify possible differences in transmission depending on the city (Abuja, Ibadan, Nnewi) or large isolate families (CAM, other modern isolates, other isolates namely *M. africanum* and *M. bovis*). The clade was found to be significantly linked to the transmission frequency as assessed by clustering, with higher transmission for T isolates (ANOVA, p = 0.012; effect sizes: “*M*. *africanum and bovis*” family = -0.25; “CAM” family = −0.01; other modern isolates [T] = +0.32). Indeed T isolates exhibited the lower proportion of orphans (59% as compared to 94% for *M. africanum* and *M. bovis* cluster and 87% for CAM). No significant statistical differences were detected regarding transmission in the different centers although a tendency for higher transmission in Abuja and Nnewi was detected (Table 3).

## Discussion

This is the largest and most detailed genetic characterisation on MTBC clinical isolates of patients suffering from TB in Nigeria relying on the analysis of isolates from three main cities [2]. The genetic diversity of MTBC was characterised by spoligotyping (43 and 68 spacers) and by 24 VNTR loci [10], [27].

Spoligotyping is a genotyping method that studies the genetic diversity of the Clustered Regularly Interspersed Palindromic Repeats (CRISPR) within the MTBC (for a review on CRISPR see [28]). It enables reliable subspecies identification [17], [29], [30]. Its recent transfer from a membrane-based to a microbead-based format resulted in a second *youth* to this method, and a similar “CRISPOL” method has recently been developed to track outbreaks for another pathogen, *Salmonella enterica* ser. typhimurium [9], [15], [31].

Increasing the number of spacers to be analysed in some settings can also improve clustering and reduce the costs of systematic Spoligo+VNTR typing as recently shown in Cambodia where the number of VNTR locus to be analysed was reduced to 8 without loss of discriminatory power [32].

We have shown in this study that the recent tuberculosis transmission rate could be between 22 and 38% using either a strict (100% identity on 24 VNTR) or smooth (including SLV) definition of clusters. We detected an active transmission of TB especially in Abuja and Nnewi, although these data need to be interpreted with caution given the short (one year) recruitment period [33]. However, looking into the social network of patients found in clusters could not be done here and is a clear limitation of this study.

The CAM genotypes were the most prevalent circulating genotypes (66%). This clade was first described in Cameroon, where it represented 34% of the *M. tuberculosis* isolates in 2003 [25]. This group of strains was assumed to have emerged recently and homogeneously in the West province of Cameroon. It is characterized by the SIT61 signature (spacers 23–25 and 33–36 missing) and a homogenous 6 bands-Ligation-Mediated PCR pattern [25]. The CAM clade belongs to the principal genetic group 2 (i.e. *modern* strains) and is lacking the TbD1 region [26]. Several CAM spoligotype variants (SIT852, SIT808, SIT403) have been reported in Cameroon and in Nigeria [26]. The 12 MIRU-VNTR signatures differ in 4 out of 12 loci (MIRU16, MIRU26, MIRU27, MIRU40) and they have similar IS*6110*-RFLP patterns (10 to 15 copies) with seven common bands [26]. This group was also shown not to have an IS*6110* copy in the DR locus and four IS*6110* copies in open reading frames coding for adenylate cyclase, phospholipase C, *moeY*, and ATP-binding proteins [26]. In rare cases, strains with identical IS*6110*-RFLP patterns had spoligotypes differing by as much as 15 spacers [26].

In a recent study, four clinical isolates belonging to the Cameroon clade were partially sequenced to detect single nucleotide polymorphisms (SNPs) and in an attempt to find new markers for molecular evolution and epidemiology. A specific non-synonymous mutation in the *dnaQ* gene was found in these four isolates of the CAM clade [34]. Whether this SNP could be used to specifically identify the CAM clade remains to be studied on a large sample in West Africa. The CAM clade had formerly been designated as a subclade of the LAM clade (LAM10-CAM) based on the common absence of spacers 23–24 [17]. However Dos Vultos and colleagues demonstrated that this clade has nothing to do with *bona fide* LAM, since it does not carry the LAM-specific SNPs [34].

In addition to Cameroon, a high prevalence of the CAM clade had been previously observed in neighbouring countries such as Chad (33%), Burkina Faso (30%) and Ghana (45%) [14], [25], [35]–[37] and a study on the genetic diversity of TB in Jos, Plateau state (Nigeria) suggested a frequency of this clade similar to the one found here [3]. Thus our study indicates that the CAM clade has a very high prevalence in Nigeria and suggests that Nigeria is the present largest reservoir for this genetic family in Africa.

*M. africanum* remains an important cause of TB in humans. Its presence in every setting of this study confirms that it is still transmitting in Africa. Its capacity to spread and to cause disease seems restricted though [38]. Here, the lower frequency of recent transmission was further documented for subfamily *M. africanum* West African 1 as all *M. africanum* isolates but one in Abuja belonged to this type [21]. Its continuing presence could however be due to different transmission dynamics, namely the ability to perform efficient retarded transmission. This possibility could be investigated using long-term molecular epidemiological studies.

Four Spoligotype profiles characteristic of *M. bovis* isolates were found in Abuja, which is in the Central North area where the population owns large herds of cattle. As in a previous study all belonged to the *M. bovis* Afri1 family that was found to infect human, cattle, goat and pigs [5]. Another former study of 55 isolates from human samples in Ibadan (South-West) revealed 11% of *M. bovis* Afri1 and zero Afri2 [39].

Regarding transmissibility of the different families, the fact that the CAM clade is very prevalent is an indirect evidence of a high fitness. VNTR3690 (Mtub39) copy number was very variable in this clade. Mtub39 is located in the promoter of the *lpdA* gene, a potential virulence factor. The number of repetitions in Mtub39 was found correlated to the expression level of *lpdA* [22].

Patho-physiological grounds on the success of the CAM clade could also be linked to the polymorphism of the 3R genes. Until now, we have no evidence of such a link although it is likely that 3R genes are major players of molecular adaptation and evolution [40], [41]. Alternatively, the main parameter responsible for the fitness of the CAM clade may be the demographic changes of the Nigerian population with a population of around 150 million in 2012 and a projected 250 million population in 2035.

Even though we did not observe strong geographical differences in the prevalence of the clades in the three cities, further analysis of data stratified by language and ethnic/tribe group and a more thorough spatial analysis may allow to better investigate bacterial genotypes-human hosts associations. Further work to characterise the phenotypic/genotyping links within *M. tuberculosis* strains circulating in Nigeria is also needed, especially on the critical issue of MDR-XDR-TB control. In this sense, new studies that will use integrated molecular methods, aimed at both MDR-TB prevention, outbreak surveillance and patient care should be implemented in Nigeria in a near future.

## Supporting Information

Table S1 **Excel File with Full Experimental Results (Sheet 1: data), Cameroon clade results (Sheet 2: CAM) and Statistics (Sheet 3: analyses).**
(XLSX)
